# Follow up of a robust meta-signature to identify Zika virus infection in *Aedes aegypti*: another brick in the wall

**DOI:** 10.1590/0074-02760180053

**Published:** 2018-05-28

**Authors:** Eduardo Fukutani, Moreno Rodrigues, José Irahe Kasprzykowski, Cintia Figueiredo de Araujo, Alexandre Rossi Paschoal, Pablo Ivan Pereira Ramos, Kiyoshi Ferreira Fukutani, Artur Trancoso Lopo de Queiroz

**Affiliations:** 1 Fundação Oswaldo Cruz-Fiocruz, Instituto Gonçalo Moniz, Salvador, BA, Brasil; 2 Fundação Oswaldo Cruz-Fiocruz, Programa de Pós-Graduação em Biotecnologia em Saúde Investigativa, Salvador, BA, Brasil; 3 Universidade Federal da Bahia, Serviço de Imunologia, Salvador, BA, Brasil; 4 Universidade Tecnológica Federal do Paraná, Cornélio Procópio, PR, Brasil; 5 Universidade de São Paulo, Faculdade de Medicina de Ribeirão Preto, Ribeirão Preto, SP, Brasil; 6 Universidade Salvador, Salvador, BA, Brasil

**Keywords:** RNA-seq, signature, transcriptome, Zika virus

## Abstract

The mosquito *Aedes aegypti* is the main vector of several arthropod-borne diseases that have global impacts. In a previous meta-analysis, our group identified a vector gene set containing 110 genes strongly associated with infections of dengue, West Nile and yellow fever viruses. Of these 110 genes, four genes allowed a highly accurate classification of infected status. More recently, a new study of *Ae. aegypti* infected with Zika virus (ZIKV) was published, providing new data to investigate whether this “infection” gene set is also altered during a ZIKV infection. Our hypothesis is that the infection-associated signature may also serve as a proxy to classify the ZIKV infection in the vector. Raw data associated with the NCBI/BioProject were downloaded and re-analysed. A total of 18 paired-end replicates corresponding to three ZIKV-infected samples and three controls were included in this study. The nMDS technique with a logistic regression was used to obtain the probabilities of belonging to a given class. Thus, to compare both gene sets, we used the area under the curve and performed a comparison using the bootstrap method. Our meta-signature was able to separate the infected mosquitoes from the controls with good predictive power to classify the Zika-infected mosquitoes.

The mosquito *Aedes aegypti* (L.) is the main vector of several globally distributed diseases (Lorenzo et al. 2014). One of these diseases is dengue (DENV) that affects more than 2.5 billion people (WHO 2015). Moreover, other illnesses such as yellow fever (YFV) are endemic to tropical regions (Bae et al. 2005) and have recently re-emerged in close proximity to major urban centres in Brazil (Paules and Fauci 2017), while West Nile fever (WNF), which usually is associated with small outbreaks, presents high mortality rates (Pradier et al. 2012). Chikungunya virus (CHIKV), once localised to parts of Africa, has now spread globally (Cauchemez et al. 2014). Zika virus (ZIKV) emerged in 2015 in the Americas (Zanluca et al. 2015, Faria et al. 2016, Slavov et al. 2016) following sporadic outbreaks in the Pacific in 2007 (Micronesian Island Yap) and 2013-14 (French Polynesia). Initially, it was not considered dangerous as it is a self-limiting disease, but later, it was associated with a ‘microcephaly outbreak’ (Tang et al. 2016), leading Brazilian authorities to declare a national health state of emergency and the World Health Organization to designate the Zika epidemic as a public health emergency of international concern (WHO 2016).

Previous studies were performed to elucidate altered pathways of *Ae. aegypti* in response to viral infections, and our group recently identified a viral infection meta-signature for DENV, WNV and YFV by investigating the relationship between feeding and infection (Fukutani et al. 2018). We identified a set of 110 genes highly correlated with viral infection, of which four genes (AAEL012128, AAEL014210, AAEL002477, and AAEL005350) were highly informative in identifying the infection-status classification in the vector. The role of the genes AAEL014210, AAEL002477 and AAEL005350 is regulatory based on the prediction of a zinc finger, DNA-binding domain (InterPro accession no. IPR013087), and basic-leucine zipper domain (InterPro accession no. IPR004827), and these genes harbour retinaldehyde binding and alpha-tocopherol transport domains (InterPro accession nos. IPR001071 and IPR001251, respectively). The gene AAEL012128 is described as having a 12-pass transmembrane protein with a cationic amino acid transporter and was previously reported as a retrovirus receptor (Wang et al. 1991, Fukutani et al. 2018).

The datasets used in the previous studies lacked ZIKV-infected samples, which were unavailable at the time. Recently, a next-generation sequencing study with *Ae. aegypti* infected with ZIKV was published (Etebari et al. 2017) with a new data, allowing a test of our signature in this infection. Our hypothesis is that the expression of signature genes, as we have demonstrated for other viruses, also plays an important role in the classification of the ZIKV infection in the mosquito. To achieve this study, we assessed the NCBI/BioProject PRJNA399504 (https://www.ncbi.nlm.nih.gov/bioproject/399504) and downloaded all the raw data. In total, there are 18 paired-end replicates corresponding to six samples (three ZIKV-infected and three controls) (Etebari et al. 2017). The fastq-dump from the SRA toolkit (NCBI 2011) was used to obtain sequence files in FASTQ format. Sequences were filtered for low quality reads and adapters using Trimmomatic version 0.32 GNU (Bolger et al. 2014), and transcripts were quantified using the *Ae. aegypti* reference transcriptome AaegL3.5 as a reference within Salmon v0.9.1 (Patro et al. 2017). Transcripts were summarised at the gene-level using the R package *tximport* (Soneson et al. 2015), yielding a count table. The count table was filtered using the *edgeR* package (Robinson et al. 2010), and only genes consistently expressed [counts per million (cpm) greater than 0.5] were kept. Each sample-expression value was determined by its sample-replicates mean, as specified by the BioProject metadata. To test our signature, a nonmetric multidimensional scaling (NMDS) was performed using the *metaMDS* function within the R package *vegan* v2.4.5 (Oksanen et al. 2017). *metaMDS* aims to represent the position of samples in a multidimensional space, as accurately as possible, using a reduced number of dimensions (axes). The axes resulting from metaMDS were used in a logistic regression model to access the predicted probability of a sample belonging to a given class (i.e., ZIKV- or mock-infected). To compare both models (the complete set of 110 genes or the restricted set of four genes), the area under the receiver operating characteristic (ROC) curve (AUC) was calculated using the *pROC* package (Robin et al. 2011).

First, we tested our previously identified signature composed of 110 genes. This set consists of the previously correlated genes with infection without the blood-feeding influence (Fukutani et al. 2018). The nMDS model was able to discriminate the groups (infected mosquitoes with ZIKV and uninfected samples) (Fig. 1A). The same approach was used with the smaller gene set (four genes) (Fig. 1B). Both the 110 and four gene sets were able to discriminate the ZIKV-infected from the uninfected samples with 7 x 10^-2^ and 2 x 10^-4^ stress values, respectively (lower stress values indicate a more reliable ordination of the dataset). To measure the accuracy of each gene set, we calculated the area under curve (AUC); using the 110 gene dataset, an AUC of 0.94 was found, whereas using a four gene dataset, the value was 0.83 (Fig. 2). However, there is no significant difference between both the AUC of both gene sets (D = -1.48, boot.n = 2000, p-value = 0.13). Despite this result, our original meta-signature was identified for the mosquito classification infected with YFV, WNF and DENV, and when applied in ZIKV-infected mosquitoes, these genes were able to discriminate the infected and healthy samples. In addition, irrespective of the set of genes selected (the 110 or four gene set), the signature remained robust with good predictive power for the classification of the Zika infection.

**Fig. 1:**
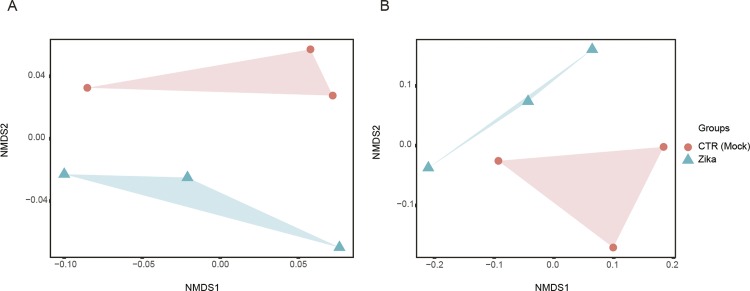
: nonmetric multidimensional scaling analysis (NMDS) based on the Bray dissimilarity index from the 110 gene set (A) and four gene set (B) to discriminate infected (blue triangles) and uninfected mosquitoes (red circles).

**Fig. 2:**
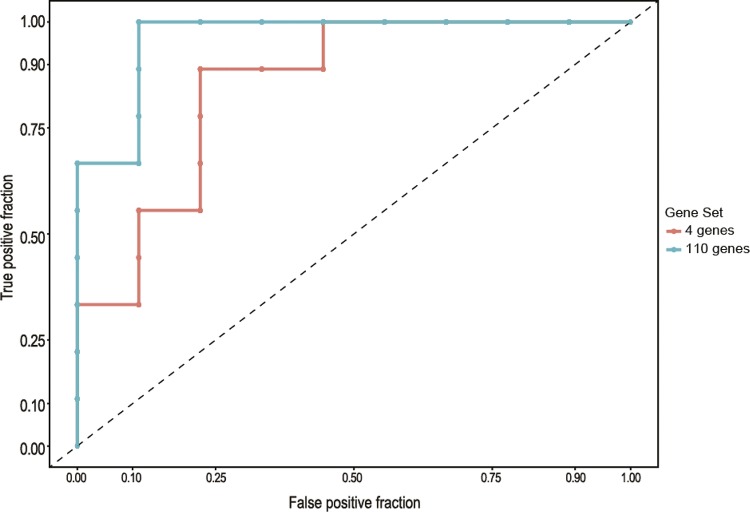
: receiver operating characteristic (ROC) curve for the gene sets. The area under the curve (AUC) for predicting Zika virus infection was 0.94 for the 110 gene (blue) set and 0.83 for the four gene (red) set.

The limitation of the current approach is the relatively low number of samples re-analysed (n = 6). However, there are few datasets available of mosquitoes infected with the ZIKV. Another available dataset of *Ae. aegypti* (GSE96605) does not have an adequate number of samples (three samples: one DENV, one ZIKV and one control with three technical replicates) (Angleró-Rodríguez et al. 2017). Despite this limitation, our results showed that these gene sets are a powerful framework for future studies of vector infections. Applications of this meta-signature are promising and suggest a process that could be similar in other vector infections such as CHIKV and Oropouche virus. However, to date, there are no publicly available datasets of vectors infected with these viruses. These results improve the predictive power of our previously identified meta-signature as a consistent signature that is able to identify mosquitoes infected by DENV, YFV, WNF and now ZIKV. This result suggests common processes are involved in all infections. Moreover, these processes impact vector pathways related to the maintenance of virus replication, such as host protein machinery and amino acid transportation processes (Mosso et al. 2010).
